# A Case Report of Spontaneous Subcutaneous Emphysema Secondary to Pulmonary Tuberculosis

**DOI:** 10.7759/cureus.82353

**Published:** 2025-04-16

**Authors:** Monika Singh, Mohit Vaid

**Affiliations:** 1 Department of Internal Medicine, Vardhman Mahavir Medical College (VMMC) and Safdarjung Hospital, Delhi, IND

**Keywords:** active tuberculosis, anti tubercular therapy, pneumomediastinum, pneumothorax, pulmonary tuberculosis, subcutaneous emphysema, tuberculosis

## Abstract

Spontaneous subcutaneous emphysema (SCE) is an uncommon clinical finding in pulmonary tuberculosis (TB) and can lead to diagnostic uncertainty due to its unusual presentation. It typically results from air leakage through cavitary lesions or bronchopleural fistulae, leading to the accumulation of air in subcutaneous tissues, often accompanied by pneumothorax and pneumomediastinum.

We report the case of a 30-year-old male with a prior history of treated pulmonary TB who presented with fever, productive cough, progressive dyspnea, and swelling of the face, neck, and chest. Examination revealed widespread crepitus over the upper body and hypoxia requiring high-flow oxygen support. Imaging showed extensive fibrocavitatory changes in both lungs, bilateral pneumothorax, pneumomediastinum, and significant SCE. Sputum acid-fast bacilli (AFB) testing was positive (3+) for *Mycobacterium tuberculosis*, and *Pseudomonas aeruginosa* was also isolated. The patient was initially started on broad-spectrum antibiotics without improvement. On Day 4 of admission, antitubercular therapy (ATT) was initiated following microbiological confirmation.

The patient showed significant clinical improvement within 72 hours of starting ATT. The SCE resolved progressively, respiratory distress diminished, and oxygen support was discontinued by Day 7. He was discharged in stable condition on Day 9 with continuation of ATT and outpatient follow-up.

This case highlights the importance of considering TB in the differential diagnosis of spontaneous SCE, particularly in endemic regions. Early recognition and initiation of ATT can result in rapid clinical improvement and prevent serious complications.

## Introduction

Subcutaneous emphysema (SCE) is defined as the presence of gas or air in the subcutaneous tissue, most often of the face, neck, and thorax. It typically arises secondary to trauma, barotrauma, surgical procedures, or underlying lung pathology that disrupts the integrity of the airways or alveoli. Spontaneous SCE, in the absence of external trauma, is uncommon and may result from infections, malignancies, or spontaneous alveolar rupture [1]. Pulmonary tuberculosis (TB), especially when associated with cavitary lesions or bronchopleural fistula, has been rarely implicated in the development of spontaneous SCE [2].

TB remains a major public health concern in India and other endemic regions, with varied clinical presentations ranging from asymptomatic infection to severe respiratory failure [3]. TB-related cavitary lesions can serve as a source of air leak into the pleural space, mediastinum, or soft tissues. The presence of SCE as a presenting symptom of active TB is rare and may obscure the diagnosis due to its non-specific and sometimes misleading clinical appearance [4]. We report a case of extensive SCE associated with fibrocavitatory pulmonary TB complicated by pneumothorax and pneumomediastinum, in a previously treated patient.

## Case presentation

A 30-year-old male presented to the emergency department with complaints of moderate-grade, intermittent fever with an evening rise in temperature, and mucopurulent sputum production for one week. He also reported progressively worsening shortness of breath over five days and new-onset diffuse swelling over the face, neck, and upper chest for the past three days. There was no history of trauma, recent surgery, mechanical ventilation, or dental procedures. The patient denied chest pain or hemoptysis. He was a non-smoker with no known history of chronic obstructive pulmonary disease (COPD). His past medical history included a prior episode of pulmonary TB several years earlier, for which he had completed a full six-month course of antitubercular therapy (ATT) (isoniazid, rifampicin, pyrazinamide, and ethambutol) with documented clinical resolution. No follow-up imaging from the previous episode was available.

**Figure 1:**
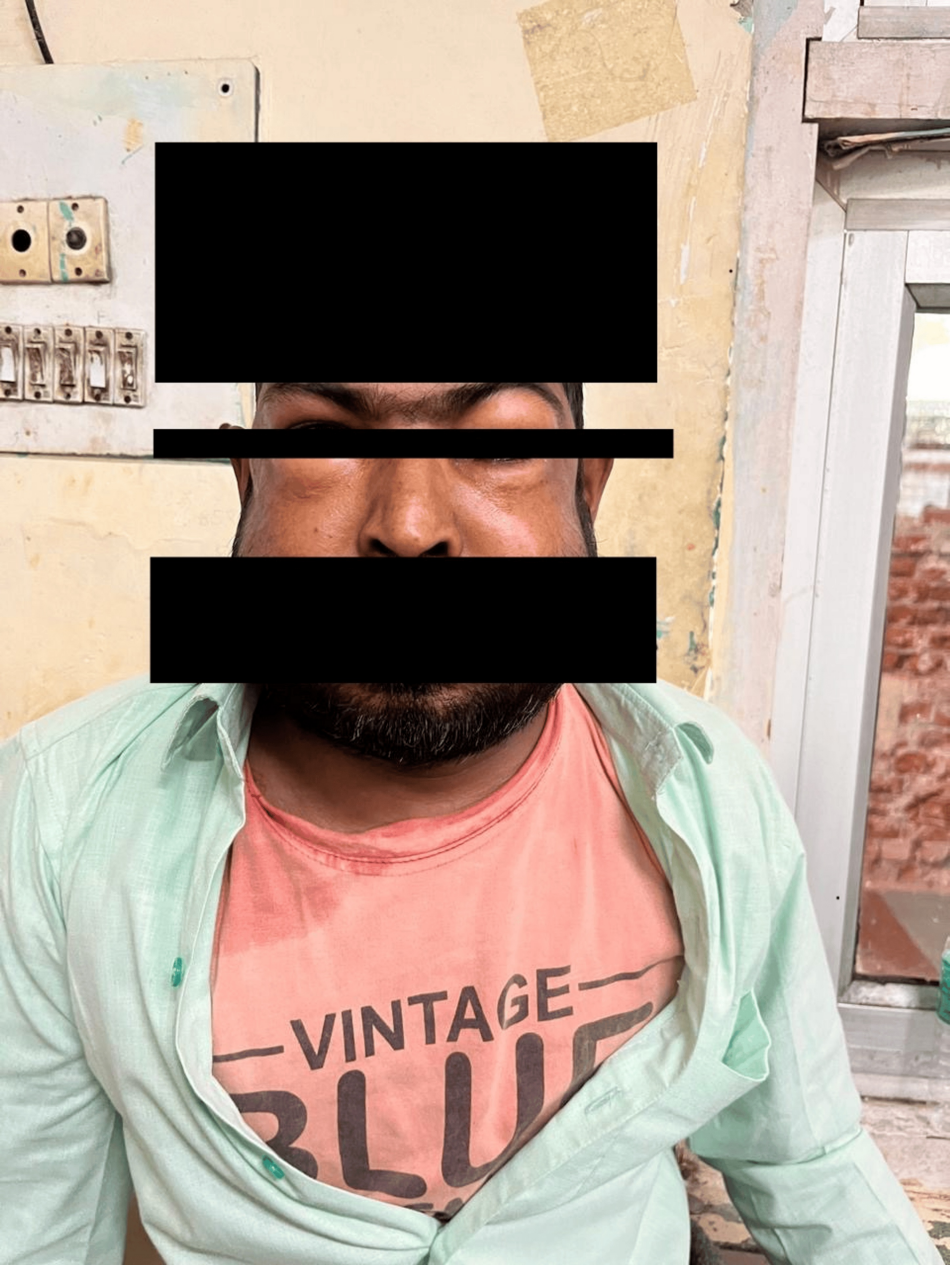
Diffuse swelling over the periorbital region and face.

On presentation, he appeared acutely unwell and dyspneic, with a respiratory rate of 32 breaths per minute, pulse rate of 106 bpm, blood pressure of 116/76 mmHg, and oxygen saturation of 84% on room air, improving to 94% on high-flow oxygen. Auscultation revealed bilateral coarse crackles with diminished breath sounds in the upper zones. No tracheal deviation was observed, and vocal resonance was normal. Diffuse swelling of the periorbital region, face, neck, and anterior chest wall was noted (Figure 1), with palpable crepitus indicative of SCE.

Initial laboratory investigations revealed a total leukocyte count of 15,600/mm³, an elevated erythrocyte sedimentation rate (ESR) of 76 mm/hour, and a raised C-reactive protein (CRP) level of 48 mg/L. Arterial blood gas on room air revealed PaO₂ of 58 mmHg, yielding a PaO₂/FiO₂ ratio of approximately 276, consistent with mild hypoxemia. Liver and kidney function tests were found to be under normal limits.

**Figure 2:**
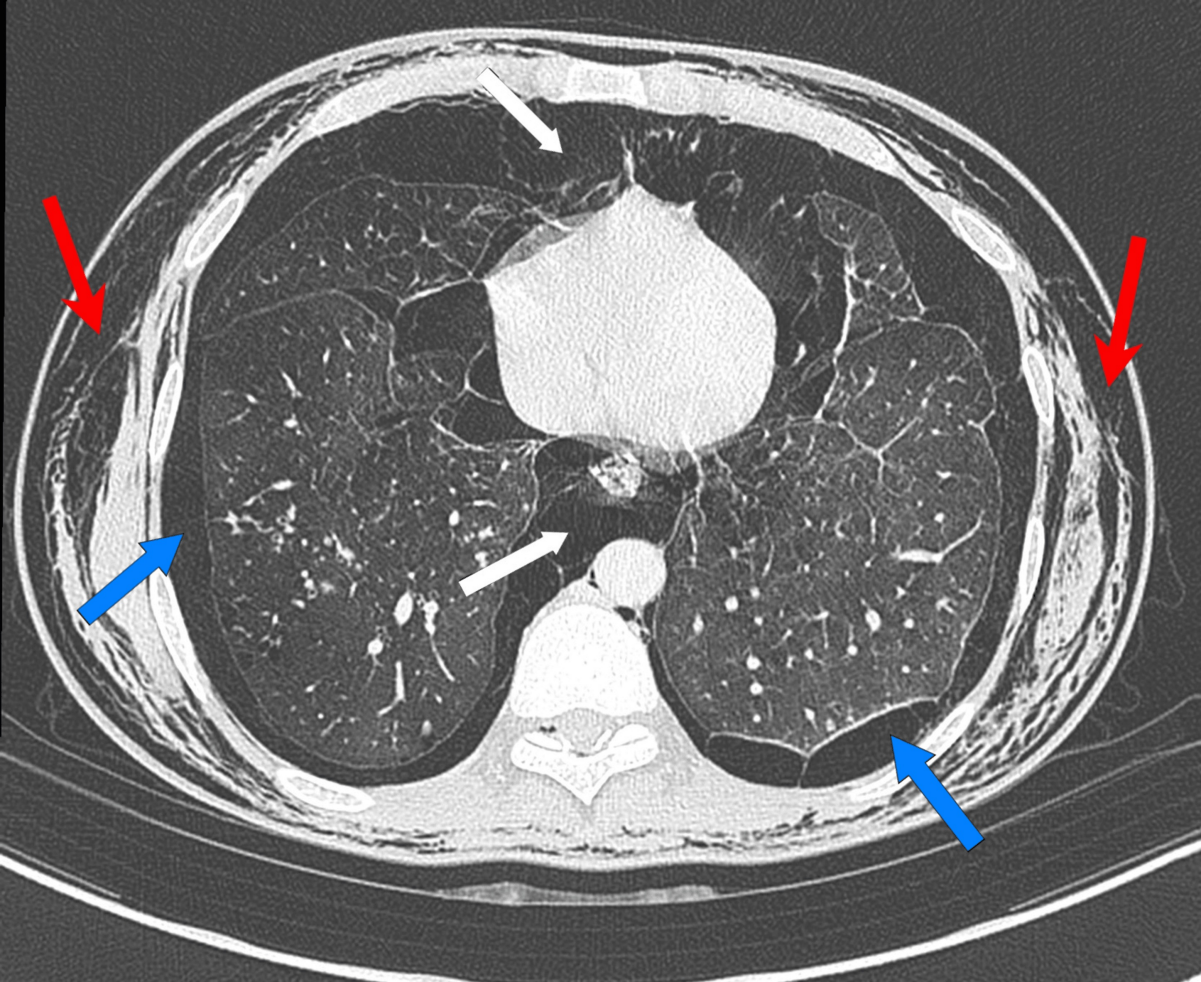
CECT chest scan of the chest revealed extensive fibrocavitatory disease, bilateral pneumothorax, pneumomediastinum, and widespread subcutaneous emphysema. CECT: contrast-enhanced computed tomography Red Arrows: Subcutaneous Emphysema; Blue Arrows: Bilateral Pneumothorax; White Arrows: Pneumomediastinum

A chest X-ray demonstrated bilateral upper zone cavitary lesions, patchy consolidates, pneumomediastinum, and soft tissue gas suggestive of SCE. A contrast-enhanced computed tomography (CECT) scan of the chest revealed extensive fibrocavitatory disease involving both upper lobes, bilateral pneumothorax, pneumomediastinum, and widespread SCE (Figure 2). A possible bronchopleural fistula was identified. Additional findings included centrilobular nodules with a tree-in-bud appearance, bronchiectatic wall thickening, and mediastinal lymphadenopathy - all supportive of active pulmonary tuberculosis.

The patient was empirically started on intravenous piperacillin-tazobactam and levofloxacin to provide dual coverage for potential *Pseudomonas aeruginosa*, considering his prior pulmonary damage. He was placed on a Venturi mask at 8 L/min with FiO₂ 50%, which was escalated to 12 L/min due to persistent hypoxia. Despite antibiotic therapy, his symptoms did not improve clinically or radiologically by Day 3. On Day 4, sputum testing for AFB returned strongly positive (3+), confirming active pulmonary TB. Culture also grew *P. aeruginosa*, which was interpreted as possible colonization in the context of pre-existing cavitary disease.

On Day 4 of admission, sputum smear testing for acid-fast bacilli (AFB) returned strongly positive (3+), confirming a diagnosis of active pulmonary tuberculosis. Based on these findings, first-line ATT (isoniazid, rifampicin, pyrazinamide, and ethambutol) was initiated.

**Figure 3:**
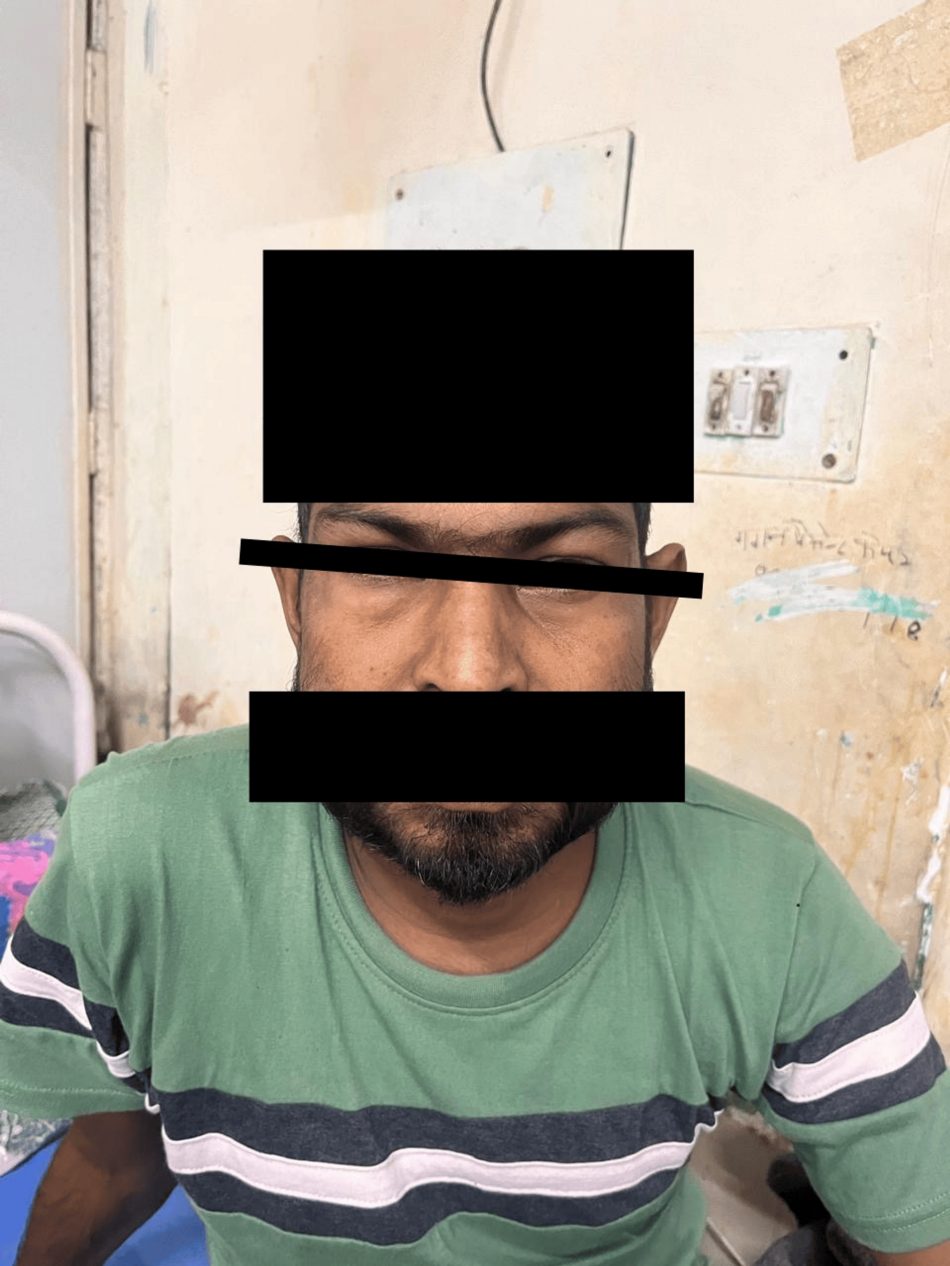
Following 72 hours of initiation of ATT, the patient’s subcutaneous swelling regressed notably. ATT: antitubercular therapy

Standard first-line ATT (isoniazid, rifampicin, pyrazinamide, and ethambutol) was initiated on Day 4. Antibiotics were discontinued after four days due to the patient’s rapid clinical improvement. Within 72 hours of ATT initiation, his respiratory distress improved significantly, the SCE regressed, and oxygen requirements decreased. By Day 7, he was off oxygen support and maintaining normal saturation on room air (Figure 3). Chest drainage was not performed, as the bilateral pneumothoraces were small and stable on serial imaging, and the bronchopleural fistula resolved without intervention.

The patient was monitored in the hospital for two more days to ensure continued improvement and was discharged on Day 9 in stable condition. He was advised to continue the full course of ATT under direct observation and scheduled for regular outpatient follow-up.

## Discussion

SCE is usually associated with traumatic or iatrogenic injury, but spontaneous cases have been described in infections such as tuberculosis, necrotizing pneumonia, and *Pneumocystis jirovecii* pneumonia (PJP) [5,6]. TB-related SCE is rare, with most literature limited to case reports and small case series. The mechanism possibly involves air tracking from a ruptured alveolus into the interstitium, progressing along the bronchovascular sheath into the mediastinum (pneumomediastinum), and subsequently into the subcutaneous tissues of the neck and chest [7].

Differentiating TB-related SCE from other causes such as cellulitis, angioedema, or necrotizing fasciitis can be challenging due to overlapping external signs. However, careful physical examination (crepitus, non-tender swelling), imaging, and microbiological testing aid in accurate diagnosis. Our patient presented with moderate-grade fever, cavitary lesions, tree-in-bud opacities, and a positive AFB smear - all pointing toward active tuberculosis.

The dramatic clinical response following the initiation of ATT, particularly the regression of SCE and improvement in respiratory parameters, supports TB as the primary etiology. While structural changes such as cavities do not reverse quickly, the reduction in air leakage and resolution of the acute inflammatory process likely led to symptom improvement. Although *P. aeruginosa* was isolated, its role appeared limited to colonization, given the lack of antibiotic improvement and rapid resolution after ATT alone.

In cavitary pulmonary TB, especially with necrotizing or bronchiectatic changes, the risk of alveolar rupture or bronchopleural fistula increases. Air may be dissected into contiguous spaces, resulting in pneumothorax, pneumomediastinum, or SCE. The simultaneous presence of all three, as seen in this case, is exceedingly rare and poses a diagnostic challenge [8]. Although small bilateral pneumothoraces and a suspected bronchopleural fistula were observed, chest tube placement was deferred in favor of conservative management. The patient’s stable condition, improving oxygenation, and absence of progression on imaging supported this decision.

Repeat CT imaging was not performed due to the patient’s rapid clinical recovery, but serial chest radiographs showed a reduction in pneumothorax and soft tissue air. The absence of prior imaging limited comparison with baseline lung architecture, but clinical correlation confirmed this was a recurrence rather than a sequela of prior disease.

Management of TB-related SCE focuses on treating the underlying infection and providing supportive care. While some patients may require surgical intervention, chest tube insertion, or drainage of pneumothorax, our patient improved without invasive measures. Finally, while the patient denied known TB contacts, reinfection remains plausible in endemic settings and prompt initiation of ATT is crucial, especially in TB-endemic regions, to avoid disease progression and complications [9,10].

## Conclusions

Spontaneous SCE is an uncommon but important presentation of active pulmonary tuberculosis. Clinicians should maintain a high degree of suspicion when evaluating patients from TB-endemic regions who present with unexplained swelling of the upper body. Detailed imaging and microbiological confirmation are essential for early diagnosis. In most cases, timely initiation of ATT leads to rapid resolution of symptoms and prevents further complications. This case contributes to the limited but growing body of literature emphasizing the atypical manifestations of tuberculosis.
